# Removing unwanted variation between samples in Hi-C experiments

**DOI:** 10.1093/bib/bbae217

**Published:** 2024-05-06

**Authors:** Kipper Fletez-Brant, Yunjiang Qiu, David U Gorkin, Ming Hu, Kasper D Hansen

**Affiliations:** McKusick-Nathans Institute of Genetic Medicine, Johns Hopkins School of Medicine, Baltimore, MD 21205, USA; Department of Biostatistics, Johns Hopkins Bloomberg School of Public Health, Baltmore, MD 21205, USA; Bioinformatics and Systems Biology Graduate Program, University of California, San Diego, La Jolla, CA 92093, USA; Ludwig Institute for Cancer Research, New York, NY 10016, USA; Ludwig Institute for Cancer Research, New York, NY 10016, USA; Department of Cellular and Molecular Medicine, University of California at San Diego, La Jolla, CA 92093, USA; Currently: Department of Biology. Emory University. Atlanta, GA 30322, USA; Department of Quantitative Health Sciences, Lerner Research Institute, Cleveland Clinic Foundation, Cleveland, OH 44196, USA; McKusick-Nathans Institute of Genetic Medicine, Johns Hopkins School of Medicine, Baltimore, MD 21205, USA; Department of Biostatistics, Johns Hopkins Bloomberg School of Public Health, Baltmore, MD 21205, USA

**Keywords:** Hi-C, bioinformatics

## Abstract

Hi-C data are commonly normalized using single sample processing methods, with focus on comparisons between regions within a given contact map. Here, we aim to compare contact maps across different samples. We demonstrate that unwanted variation, of likely technical origin, is present in Hi-C data with replicates from different individuals, and that properties of this unwanted variation change across the contact map. We present band-wise normalization and batch correction, a method for normalization and batch correction of Hi-C data and show that it substantially improves comparisons across samples, including in a quantitative trait loci analysis as well as differential enrichment across cell types.

## INTRODUCTION

The Hi-C assay allows for genome-wide measurements of chromatin interactions between different genomic regions [1–5]. The use of Hi-C has revealed that the genome is organized in structures at different resolutions such as A/B compartments [1], topologically associated domains (TADs) [6–8] and loops [9]. Our recent work [10] has also demonstrated inter-subject variability in these structures.

In addition to large-scale structures such as TADs and A/B compartments, there is substantial interest in using Hi-C data to measure specific interactions such as those occurring between regulatory elements and their associated promoters. These interactions are represented as individual cells in the Hi-C contact matrix. Such regulatory interactions do not occur at all distances; an example is enhancer–promoter contacts, which are thought to occur primarily within 1 Mb [11]. Methods for detecting such interactions include Fit-HiC [12] and HiC-DC [13]; these methods compare specific contact cells with a background distribution.

Variation and noise in an Hi-C experiment can differ between resolutions and between different types of structures. For example, A/B compartments are estimated using an Eigen decomposition of a suitably normalized contact matrix. We have previously [14] found little-to-no differences between A/B compartments estimated using data from a 1 Mb resolution dilution Hi-C experiment [1] and a 1 Kb resolution *in situ* Hi-C experiment on the same cell line [9]. This observation is specific to A/B compartments; the two experiments differ dramatically in terms of resolution and ability to estimate many other types of structures including TADs and loops.

Hi-C data, like all types of genomic data, suffer from systematic noise and bias. To address this, a number of within-sample normalization methods have been developed. Some of these methods explicitly model sources of unwanted variation, such as GC content of interaction loci, fragment length, mappability and copy number [15–17]. Other methods are agnostic to sources of bias and attempt to balance the marginal distribution of contacts [9, 18–20]. A comparison of some of these methods found high correlation between their correction factors [9].

When comparing genomic data *between* samples, variation can arise from numerous sources that do not reflect the biology of interest including sample procurement, sample storage, library preparation and sequencing. We refer to these sources of variation as ‘unwanted’ here, because they obscure the underlying biology that is of interest when performing a between-sample comparison. It is critical to correct for this unwanted variation in analysis [21]. A number of tools and extensions have been successful at this, particularly for analysis of gene expression data [22–28]. Most existing normalization methods for Hi-C data are single sample methods, focused on comparisons between different loci in the genome.

Three existing methods have considered between-sample normalization in the context of a differential comparison (diffHiC, HiCcompare and multiHiCcompare [29–31]); all can be viewed as an adaption of the idea of loess normalization from gene expression microarrays [32]. In these methods, the estimated fold-change between conditions are modeled using a loess smoother as a function of either average contact strength [29] or distance between loci [30, 31]. Using the loess estimates, the data are corrected so there is no effect of the covariate on the fold-change.

## RESULTS

###  

#### High-quality Hi-C experiments on different individuals

To investigate the variation between Hi-C data generated from individuals with different genetics, we use existing dilution Hi-C data from lymphoblastoid cell lines generated from eight different individuals (including two trios) from the HapMap project [33] (Table 1). The individuals cover three populations (Yoruba, Han Chinese and Puerto Rico). For each individual, data were generated from two cultures of the same cell line grown separately for at least two passages, and more than 500 million mapped reads were generated for each individual (Table 2); at least 250 million reads for each growth replicate. The reads were summarized at a resolution of 40 kb.

**Table 1:** Sample information

Sample	Replicate	Ethnicity	Sex	Family	Role	Batch	Prep date	SCC
HG00512	1	CHS	M	2	Father	B1	3/4/15	0.923
HG00512	2	CHS	M	2	Father	B2	5/28/15	
HG00513	1	CHS	F	2	Mother	B1	3/4/15	0.961
HG00513	2	CHS	F	2	Mother	B2	5/28/15	
HG00514	1	CHS	F	2	Child	B1	3/4/15	0.967
HG00514	2	CHS	F	2	Child	B2	5/28/15	
HG00731	1	PUR	M	3	Father	B1	3/4/15	0.963
HG00731	2	PUR	M	3	Father	B2	5/28/15	
HG00732	1	PUR	F	3	Mother	B1	3/4/15	0.956
HG00732	2	PUR	F	3	Mother	B2	5/28/15	
HG00733	1	PUR	F	3	Child	B1	3/4/15	0.971
HG00733	2	PUR	F	3	Child	B2	5/28/15	
GM19238	1	YRI	F	1	Mother	B3	9/26/14	0.973
GM19238	2	YRI	F	1	Mother	B3	9/26/14	
GM19239	2	YRI	M	1	Father	B3	9/26/14	N/A

**Table 2:** Mapping statistics

Sample	Replicate	Total Reads	Cis	Cis (Long)	Trans
GM19238	1	545 759 860	302 092 644	230 613 702	243 667 216
GM19238	2	314 967 258	185 913 678	145 343 000	129 053 580
GM19239	2	553 838 876	367 216 970	287 593 654	186 621 906
HG00512	1	311 906 326	139 566 774	94 984 622	172 339 552
HG00512	2	270 228 888	152 292 628	114 914 390	117 936 260
HG00513	1	371 772 886	174 783 850	125 704 946	196 989 036
HG00513	2	277 954 128	161 423 552	122 711 298	116 530 576
HG00514	1	354 765 444	210 846 240	103 777 676	143 919 204
HG00514	2	266 032 734	177 325 378	100 665 340	88 707 356
HG00731	1	324 496 352	173 380 026	105 098 564	151 116 326
HG00731	2	266 661 686	151 763 346	99 399 932	114 898 340
HG00732	1	419 151 786	237 460 978	117 332 062	181 690 808
HG00732	2	291 561 824	176 279 418	99 373 490	115 282 406
HG00733	1	356 662 684	185 558 600	112 732 352	171 104 084
HG00733	2	293 167 014	178 562 640	100 250 886	114 604 374

Quality control using recently developed guidelines [34] suggests that our data are of high quality. In support of this conclusion, we used HiCRep to compute stratum-adjusted correlation coefficients (SCCs) between replicates of the same cell line [35]. This shows a minimal between-growth-replicate SCC of 0.92 with a mean of 0.96, comfortably exceeding the values recommended by Yardımcı *et al*. [34].

In our experimental setup, replicate 1 of GM12239 was prepared in a batch separate from all other samples. For this reason, it is hard to assess the variation within and between batch and this replicate is not included in our analysis.

###  

#### Experimental design and replication

We use lymphoblastoid cell lines from the HapMap project [33], because these cell lines have been a widely used model system to study inter-individual variation and genetic mechanisms in numerous molecular phenotypes including gene expression, chromatin accessibility, histone modification and DNA methylation [36–43]. It has been established that phenotypic differences, which are unlikely to be explained by genetics, exist between lymphoblastoid cell lines from different HapMap populations [36, 44, 45]. These differences might be related to cell line creation and division [44]. In our experimental design, experimental batch (library preparation) is partly confounded by cell line population (Table 1), because batch B3 consists solely of samples from the Yoruban population, whereas batch B1 and B2 contain one growth replicate each from the samples from the Han Chinese and Puerto Rican populations. In addition, batch B1 and B2 were prepared closer together in time (within 3 months) compared with batch B3 (6 months earlier).

To avoid confusion in the present manuscript, we will use the term ‘individual replicate’ to refer to a replicate experiment performed on lymphoblastoid cells lines created from two distinct individuals. And we will use the term ‘growth replicate’ to refer to a replicate experiment on a different growth of the same cell line—this is what is commonly referred to as a ‘biological replicate’ in the Hi-C literature (see the ENCODE Terms and Definitions https://www.encodeproject.org/data-standards/terms/).

###  

#### Unwanted variation in Hi-C data varies between distance stratum

**Figure 1:**
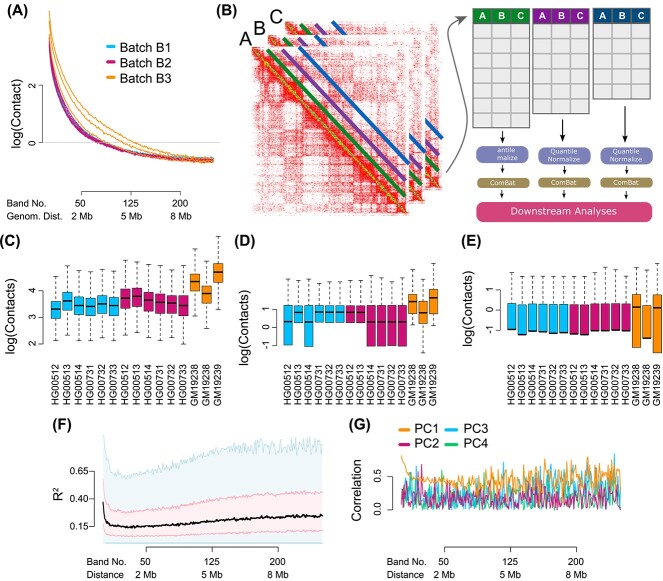
**Substantial between-sample variation in unnormalized Hi-C data.** We display Hi-C data from chromosome 14 from eight different individuals, seven of which have two technical replicates, processed in three batches. The data have not been normalized apart from correction for library size using the log counts per million transformation; data are on a logarithmic scale. (**A**) Mean contact as a function of distance. Each sample is a separate curve. (**B**) Band transformation of a collection of Hi-C contact maps. Example data are Hi-C matrices from samples A, B, C. The green, purple and blue diagonal lines represent loci separated by different distances $d_{i}$. For the $i$-th distance between loci, we group all matrix cells from each sample into a matrix whose rows are Hi-C contact cells and columns are samples. In the figure these matrices are colored by the green, purple and blue lines to indicate which distance $d_{i}$ the matrix represents; we refer to these matrices as ‘band matrices’. We apply quantile normalization followed by ComBat to each distance matrix separately. The resulting batch effect-corrected matrices can be used in downstream applications. (**C**)–(**E**) Boxplots of the marginal distribution of contacts across samples, for loci separated by (**C**) 40 kb (band 2), (**D**) 2 Mb (band 50) and (**E**) 8 Mb (band 200). (**F**) The percentage of variation explained ($R^{2}$) in a linear mixed effect model with library preparation as explanatory variable. The plot is a smoothed boxplot (Methods) with the black line depicting the median, the red shape depicting the 25%- and 75%-quantiles and the blue shape depicting 1.5 times the interquartile range. (**G**) The Spearman correlation of the library preparation factor with each of the first four principal components of each band matrix.

It is well described that an Hi-C contact map exhibits an exponential decay in signal as the distance between loci increases [1]. When we quantify this behavior across growth and individual replicates, we observe substantial variation in the decay rate from sample to sample (Figure 1A). The data presented in Figure 1a are unnormalized, but they strongly suggest systematic unwanted variation[21, 24] between experimental batches; batch B3 (Yorubans) is different from the other two batches, although quality control suggests batch B3 samples are comparable with the rest.

To assess unwanted variation beyond changes in the mean, we represented our data as a set of matrices indexed by genomic distance (Figure 1B). Each matrix contains all contacts between loci at a fixed genomic distance for all samples (Methods). We call this a band transformation, since these contacts form diagonal bands in the original Hi-C contact matrices (a band in our usage is sometimes described as a matrix off-diagonal). Figure 1C–E depicts the distributions for three selected bands at close (40 kb), medium (2 Mb) and long (8 Mb) ranges. These distributions suggest that band-specific variation is also systematically different between batches.

To quantify the impact of unwanted variation on our Hi-C data, we estimate the variation attributable to experimental batches B1, B2 and B3 (see Table 1). We measure the amount of explained variation using $R^{2}$ from a linear mixed effects model with a random effect to model the increased correlation between growth replicates (Methods). We observe an association between explained variation and distance between loci (Figure 1F), with an average $R^{2}$ value of 0.23. This means that 23% of the between-sample variation in the individual entries in the contact matrix is explained by experimental batch, which is partly confounded with population (explored further below). This shows that the effect of the experimental batch factor changes with distance and is substantial.

To further explore the effect of batch, we performed principal component analysis on each of the band matrices and computed Spearman correlation between each of the first four principal components and the batch indicator (Figure 1). This supports the conclusion of our $R^{2}$ analysis and emphasizes the dynamic nature of the association between variability and the experimental batch factor.

###  

#### Observed–expected normalization between samples

A number of normalization methods have been developed for Hi-C data, many with the explicit purpose of removing bias along the genome (see the Introduction). A popular method is ICE [18]. Observed–expected normalization was introduced by Lieberman-Aiden *et al*. [1]; it consists of dividing all contact cells in a given band by the mean contact across the band. Given the differences in decay rates across samples (Figure 1A), it is natural to force the decay rates to be the same. Observed–expected normalization is an approach to this, since it removes the decay from any sample. To keep the fast decay rate in the data, we suggest multiplying the band matrices by the average decay rate (Methods). This is a natural adaptation of observed–expected normalization to a *between-samples* approach. We combine observed–expected between-sample normalization with ICE, and we refer to this combined method as ICE-OE.

**Figure 2:**
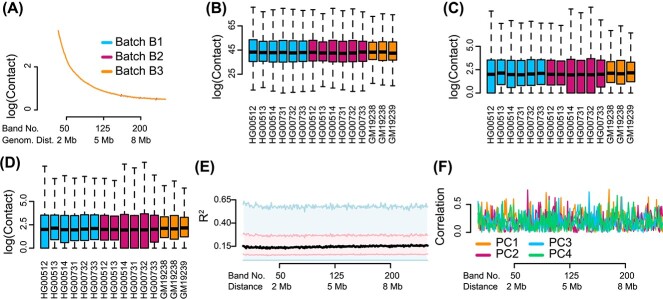
**Unwanted variation in Hi-C data normalized by ICE-OE.** Hi-C data are normalized by ICE followed by observed–expected normalization across samples (ICE-OE). (**A**) Mean contact as a function of distance. (**B**)–(**D**) Boxplots of the marginal distribution of contacts across samples, for loci separated by (**B**) 40 kb (band 2), (**C**) 2 Mb (band 50) and (**D**) 8 Mb (band 200). (**E**) The percentage of variation explained ($R^{2}$) in a linear mixed effect model with library preparation as explanatory variable. (**F**) The Spearman correlation of the library preparation factor with each of the first four principal components of each band matrix.

Using ICE-OE leads to a substantial improvement (Figure 2). Per design, there is no between sample variation in the contact decay rate. Boxplots of the contact distribution for selected bands still show sample-specific variance. More important, we no longer observe any dependence of $R^{2}$ on band, and the average $R^{2}$ is at the level of the smallest $R^{2}$ for unnormalized data (i.e. 0.15). While the $R^{2}$ is smaller than for unnormalized data alone, we note that, for each distance band, 25% of the contact cells still have an $R^{2}$ of 0.3 or greater. Likewise we observe improvement in the correlation between principal components and the batch factor. With these assessments, ICE-OE appears to have addressed many of the major deficiencies associated with unnormalized data.

**Figure 3:**
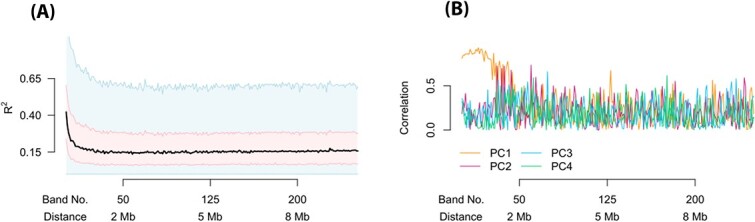
**Unwanted variation and multiHiCcompare**. Like Figures 1F and 2E, but for data normalized using multiHiCcompare.

We note that multiHiCcompare performs almost as well as ICE-OE on the evaluation of $R^{2}$ over band (Figure 3A), although compared with ICE-OE some nonlinear trend with respect to distance can be seen among loci that are separated by approximately 400 kb or less. Similarly, multiHiCcompare also largely reduces the correlation of batch effect and principal components (Figure 3), although once again loci separated by approximately 400 kb or less do continue to exhibit a relationship between distance and batch effect.

###  

#### ICE-OE is unsuitable for genetic mapping

An interesting biological question, which can only be addressed with data on individual replicates, is the association between genetic variation and 3D structure. This question can be asked for any type of 3D structure including TADs and loops. Here we focus on variation in individual contact cells, which is interesting because of the relationship between regulatory elements and the genes they regulate. Specifically, we perform a quantitative trait loci (QTL) mapping for each contact cell. A QTL mapping tests, for one contact cell, whether there is an association with a nearby single nucleotide variant (SNP). An advantage of QTL mapping is well-established quality control procedures which can help reveal whether a data matrix has been properly normalized. We recently described a complete investigation of the genetic contribution to 3D genome structure [10].

**Figure 4:**
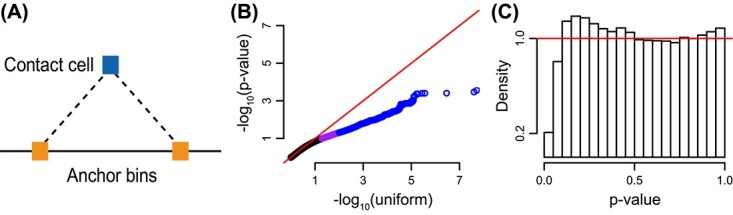
**A QTL analysis reveals incorrect normalization.** We performed a QTL analysis for association between suitably chosen SNPs on chromosome 22 and contact cells. Data were normalized using either ICE or ICE-OE. (**A**) A diagram of the search procedure. We restricted SNPs to be present in the anchor bins. (**B**) QQ-plots comparing the -$\log _{10}$*P*-value (x-axis) with $-\log _{10}$ quantiles from the uniform distribution for data normalized using ICE-OE. Colors: blue if the *P*-value is in the $99{th}$ percentile or greater, purple if the *P*-value is in the $95{th}$ to $99{th}$ percentiles and black otherwise. Note that no test has a *P*-value less than $10^{-6}$. (**C**) A histogram of the *P*-values from (A).

For QTL mapping we consider contact cells representing loci separated by less than 1 Mb. We require a candidate SNP to be present in one of the two anchor bins for that contact cell (Figure 4A, Methods), and that all genotypes are represented in our samples (Methods), resulting in 22 541 SNPs for 21 017 contact cells on chromosome 22, representing 1111 407 tests. We use a linear mixed effect model with a random effect on the growth replicate, to model the increased correlation between growth replicates.

In Figure 4 we depict a quantile–quantile plot (QQ-plot) for the (minus logarithmic) *P*-values for this analysis, as well as a histogram of the *P*-value distribution. We observe that the QQ-plots look unsatisfactory with an unusual discrepancy from expectation (parallel with the $y=x$ line with a deviation toward the end). Furthermore, the *P*-value histograms are also strongly deviating from the expected behavior of being flat with a possible bump near zero. We stress that the lack of small *P*-values revealed by the histogram is not caused by lack of power due to small sample size; this would result in a flat histogram. We conclude that ICE-OE does not properly normalize the data for a QTL analysis.

###  

#### Band-wise normalization and batch correction

To normalize the data and remove unwanted variation for a QTL analysis, we used the band transformation framework (Figure 1B). We propose to separately smooth each contact matrix, and then assemble each band matrix. Each column of the band matrix are all loci separated by the same unit of distance (so called because the columns are off-diagonal ‘bands’ in each contact matrix), one for each sample. These matrices are then quantile normalized, and finally corrected for batch effect using ComBat. We call this approach band-wise normalization and batch correction (BNBC). We next describe our rationale for each step.

We start by following existing work by Yang *et al*. [35] and smooth the sample-specific contact matrices to maximize correlation between growth replicates.

**Figure 5:**
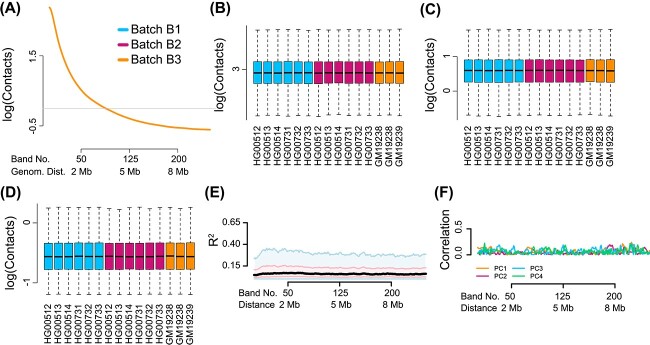
**BNBC removes unwanted variation in Hi-C data.** As Figure 2 but for data processed using BNBC. (**A**) Mean contact as a function of distance. (**B)–(D**) Boxplots of the marginal distribution of contacts across samples, for loci separated by (**B**) 40 kb (band 2), (**C**) 2 Mb (band 50) and (**D**) 8 Mb (band 200). (**E**) The percentage of variation explained ($R^{2}$) in a linear mixed effect model with library preparation as explanatory variable. (**F**) The Spearman correlation of the library preparation factor with each of the first 4 principal components of each band matrix.

We next process each smoothed matrix band, from all samples, one at a time, as smoothing has been shown to improve the reproducibility of data generated from the same subject [34]. Chromosomes are processed separately. We perform quantile normalization on each matrix band, forcing the marginal distributions of each sample to be the same (Figures 1A,C–E, 5A–D). This reduces inter-sample variability, assuming that the distribution of contacts at a given distance is the same across samples.

We then use ComBat [25] to remove the effect of batch in each band matrix separately, because the exponential decay of the contact matrices would make contact cells from different bands incomparable. By default, ComBat removes the effect of batch on both the mean and variance of a given Hi-C matrix cell’s observations across samples, using Empirical Bayes methods to regularize estimates of batch effects. This results in more stable estimates, particularly in the small-sample setting.

BNBC is highly scalable because we only process one matrix band at a time. The largest band—the diagonal—has a number of entries equal to the number of bins in the genome, and this size scales linearly with resolution. A 1kb resolution Hi-C experiment has 3M entries in its diagonal, resulting in a band matrix with 3M rows and columns equal to the number of samples. For further scalability, we process each chromosome separately. While big, this can be processed on a laptop. We provide an implementation supporting the cooler format [46].

BNBC removes any between sample difference in decay rate and also stabilizes band-specific variances across samples (Figure 5). To assess the impact of BNBC, we again measured the variation explained by the batch factor. We observe a decrease in this quantity compared with ICE-OE, including at the 75%-quantile level (Figures 2 and 5). Likewise, we observe a dramatic decrease in correlation between principal components and the batch factor. We also experimented with removing smoothing and found an increase in the variability of the correction of batch over distance (see Figure S4); we recommend users apply smoothing, although we provide the option for users who do not wish to apply smoothing.

While seemingly impressive, we note that the decrease in $R^{2}$ and the lack of correlation with principal components are mathematical consequences of the use of regression in ComBat. This is because regressing out a factor from each of the entries in a band matrix ensures that both $R^{2}$ for that factor and the correlation between factor and each of the principal components of the data matrix are equal to zero (because ComBat uses Empirical Bayes techniques to shrink the parameters and models changes in variation, the observed $R^{2}$ and the correlations are not exactly 0). For this reason, we caution against the use of these evaluation criteria for assessing the performance of BNBC, although this is not true for methods like ICE and ICE-OE which are not based on a regression model.

**Figure 6:**
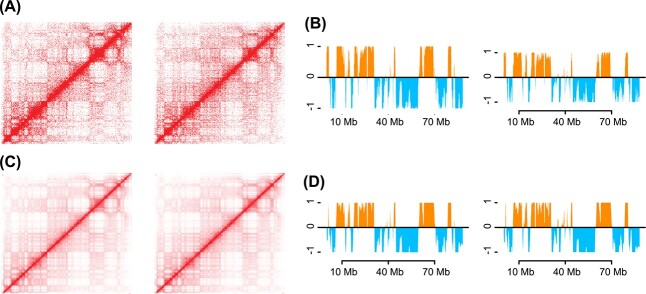
**BNBC preserves features of the contact map.** Two samples (left: GM19238, right: HG00512) from two different batches are processed using either ICE-OE or BNBC. We display data from chromosome 14. (**A**) Contact maps with data processed using ICE-OE. (**B**) A/B compartments using the first principal component of the observed–expected normalized contact matrix with data processed using ICE-OE. (**C**)–(**D**) Like (A)–(B) but with data processed using BNBC.

We next investigated the impact of BNBC on the contact map. There is little difference between the contact map following ICE normalization and BNBC normlization (Figure 6). The same is true for the associated first Eigenvector, which is commonly used to identify A/B compartments (Figure 6). The correlation between the compartment vectors obtained using BNBC and ICE-OE is 0.959 for GM19238 and 0.957 for HG00513. Further, when comparing contact matrix cells before and after BNBC, the difference is significantly less than would be expected by chance (Methods; see Figures S2 and S3). We conclude that BNBC does not distort gross features of the contact map.

**Figure 7:**
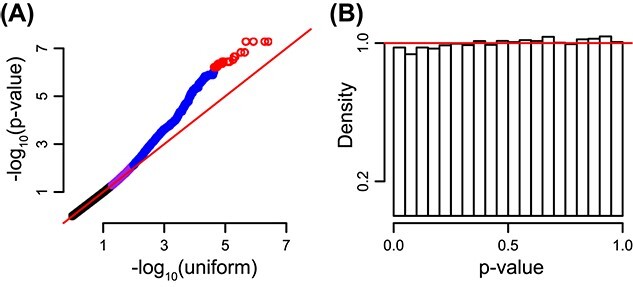
**BNBC normalizes data for QTL analysis.** Like Figure 4 but for data normalized using BNBC. (**A**) QQ plots comparing the -$\log _{10}$*P*-value (x-axis) with $-\log _{10}$ quantiles from the uniform distribution. Colors: red if the *P*-value is less than $10^{-6}$, blue if the *P*-value is in $99{th}$ percentile or greater, purple if the *P*-value is in the $95{th}$ to $99{th}$ percentiles and black otherwise. (**B**) A histogram of the *P*-values from (A).

We now consider the impact of BNBC on genetic mapping. Using the same measures as described above, we observe a uniform distribution of *P*-values as well as a much better behaved QQ-plot for the *P*-values (Figure 7). Multiple observed *P*-values are less than $10^{-}6$ (we do more than 1M tests), comfortably exceeding the lowest *P*-value following ICE or ICE-OE (which is around $10^{-4}$, Figure 4), suggesting that BNBC not only corrects issues with under-inflation of the test statistic, but also increases power. Examining QTLs with *P*-value < 1e-6 (equivalent to a 0.7% FDR under Benjamini–Hochberg correction), we find 29 genomic interaction QTLs (Table S3), which associate into seven independent loci (Figure S5). Two variants, rs2071897 and rs2071899 (P < 5.4e-8 for both), are also proximal to rs9616701, an index eQTL for *C22orf34* [47]. We conclude that BNBC noticeably improves on ICE and ICE-OE for genetic mapping.

###  

#### BNBC in TAD and loop identification

Although the motivating use case for BNBC is the comparison of individual genomic interactions (Hi-C matrix cells) across multiple subjects, the impact of batch effect correction on larger genomic structures such as loops and TADs is of interest. To assess the effect of batch effect correction on loop calling, we apply Mustache [48] (Methods) to the chr14 matrix from GM1938, replicate 1 (see Table 1). We find no loops at an FDR of 5% in data normalized by BNBC nor ICE-OE; in multiHiCcompare-normalized data we detected 13 loops along chr14 (see Figure S6, Table S2). To assess the comparability of identified TADs, we form all pairwise comparisons of samples from Table 1, and assess TAD sharing using TADCompare [49] (Methods). On average, 80% of TAD comparisons using ICE-OE-transformed data are found to agree, while 70% of comparisons from BNBC-transformed data agree. TADCompare also flags ‘complex’ comparisons wherein multiple TADs may be overlapping across samples; for ICE-OE, an average 17.8% of comparisons are ‘complex’, while an average of 27.4% of comparisons were ‘complex’ under BNBC.

###  

#### BNBC accommodates sparse contact matrices and substantial coverage variation

To demonstrate the scalability of our approach, and to evaluate BNBC on a separate dataset, we next considered the setting of a sparse, 5k-resolution contact matrix. Specifically, we consider the set of interactions on chr22 from [50], a study comparing contact propensity between induced pluripotent stem cells (iPSCs) and iPSC-derived cardiomyocytes (CMs), in seven members of a multi-generational family using *in situ* Hi-C. Here we demonstrate that BNBC is applicable to process sparse 5kb matrices and also increases power to detect contact matrix cells which are differentially enriched between cell types.

Hi-C libraries were generated at two different time points (Table 3), with the majority of libraries being generated at one time point (and a few libraries generated at a single different time point). The publicly available processed data are at the (subject, cell type) level and different samples are the result of different pooling strategies. First, some samples were generated using only a single library and some samples were generated by pooling two libraries. As all libraries were sequenced to approximately the same depth, this creates a substantial fixed difference in library size which—to a first approximation—can be explained by whether the sample was pooled or not (column ‘Pooled’ in Table 3, compared with column ‘Library Size’). Secondly, when a sample resulted as a pool of two different libraries, sometimes the two Hi-C libraries were prepared in different batches or not (column ‘MixBatch’ in Table 3). Together, these two variables (‘Pooled’ and ‘MixBatch’) creates three different levels since there are no samples that mixed batches and were not pooled, which is captured in the ‘Batch’ column of Table 3.

**Table 3:** Greenwald sample information

Sample	Cell type	MixBatch	Pooled	Batch	Library size
iPSCORE 2.1	iPSC	no	yes	B1	1646 980
iPSCORE 2.1	CM	yes	yes	B2	1490 690
iPSCORE 2.2	iPSC	no	yes	B1	1505 519
iPSCORE 2.2	CM	no	no	B3	703 848
iPSCORE 2.3	iPSC	yes	yes	B2	1718 901
iPSCORE 2.3	CM	yes	yes	B2	1707 640
iPSCORE 2.4	iPSC	no	no	B3	797 648
iPSCORE 2.4	CM	no	yes	B1	1454 185
iPSCORE 2.6	iPSC	no	no	B3	920 916
iPSCORE 2.6	CM.	no	no	B3	695 501
iPSCORE 2.7	iPSC	no	no	B3	865 444
iPSCORE 2.7	CM	no	yes	B1	146 7740
iPSCORE 2.9	iPSC	yes	yes	B2	1928 327
iPSCORE 2.9	CM	no	yes	B1	1286 331

**Figure 8:**
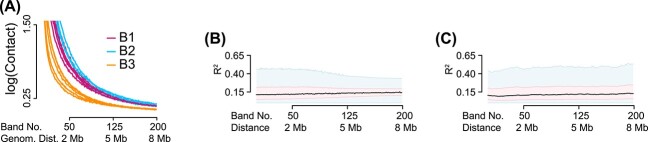
**Unwanted variation in Greenwald *et al*. data.** (**A**) Mean contact strength as a function of distance in unnnormalized data, stratified by ‘Batch’ status (Table 3). (**B**) Partial $R^{2}$ as a function of distance, for data processed with ICE-OE. (**C**) As in (B), but for data processed with BNBC.

To investigate unwanted variation, we first consider the mean contact as a function of distance (Figure 8A). As noted by Lun [51], application of standard logCPM to very sparse matrices can create statistical artifacts, although the procedure can be modified to prevent this by the use of a data-driven normalization constant (henceforth, ‘modified logCPM transformation’; see Methods). We recommend the users analyzing 5kb-resolution Hi-C data to use the modified logCPM (available in our software). In this work, we apply the modified logCPM transformation to the set of chr22 matrices and smooth each contact matrix. This demonstrates systematic variation between the three levels of ‘Batch’ (the possible combinations of ‘Pooled’ and ‘MixBatch’, see above). It may be surprising to see an effect of ‘Pooled’—this variable is largely describing variation in library size and the logCPM we plot are corrected for library size—but we hypothesize this is caused by the high sparsity of the data where a doubling in library size has a large effect on the sparsity pattern. The effect of ‘MixBatch’ is more in line with the effect we have seen in the analysis of the LCL data (above), although we stress that while we have two library preparation dates, the effect of library preparation date is mitigated by the pooling strategy.

We next compare ICE-OE and BNBC (Figure 8), and use partial $R^{2}$ to account for the variation coming from the effect of cell type in addition to batch factor. ICE-OE and BNBC appear to produce data without substantial unwanted variation by this measure, except a slight dependence of $R^{2}$ on distance for ICE-OE, and a slight increase in $R^{2}$ at long distances under BNBC.

###  

#### BNBC enhances discovery power while preserving fine-scale detail

**Figure 9:**
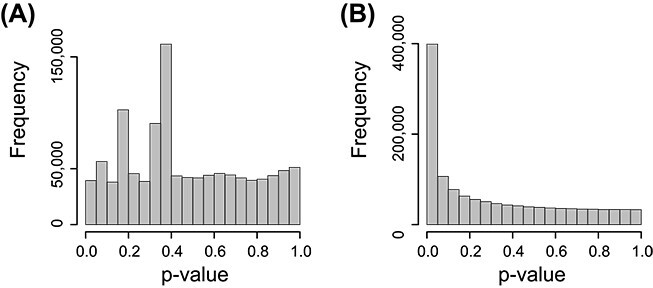
**Differential enrichment in the Greenwald data.** (**A**) *P*-value histogram from cell-type differential enrichment analysis using data processed by ICE-OE. (**B**) As (A) but for *P*-values obtained from BNBC-processed data.

To assess BNBC’s discovery power relative to ICE-OE, we conduct a differential contact analysis between cell types, among the first 200 matrix bands (see Methods). Examining Figure 9A, it is clear that ICE-OE *P*-values exhibit unusual behavior inconsistent with the assumption of null *P*-values following a uniform distribution. Additionally, there is virtually no enrichment of *P*-values closer to 0, suggesting that ICE-OE is neither calibrated nor powered to detect systematic variation across samples. By contrast, BNBC has clear enrichment of smaller *P*-values, indicating substantially improved power to detect signal (Figure 9B), with much improved calibration compared with ICE-OE.

Quantifying the utility of either method, we report the number of hits discovered (Table 4) at a family-wise error rate (FWER) of 0.05 (using a Bonferroni corrected), as well as false discovery rate (FDR) of 0.05, 0.01 and 0.001 (using independent hypothesis weighting, Methods). At no threshold does ICE-OE produce discoveries. Conversely, BNBC is well-powered to make discoveries. Considering stringent thresholds, a Bonferroni correction yields 1485 significant matrix entries, while we observe 28 354 significant matrix entries at an FDR of 0.001. We used the ICE implementation from Juicer [52] which has its own filtering steps and for that reason we perform fewer tests for ICE-OE compared with BNBC. When we restrict BNBC to the entries kept by Juicer, we get the same behavior.

**Table 4:** Number of significant tests for differential enrichment

Method	Number of tests	Bonferroni	IHW	IHW	IHW
		FWER < 0.05	FDR < 0.05	FDR < 0.01	FDR < 0.001
ICE-OE	1099 648	0	0	0	0
BNBC	1269 066	1485	229 076	104 507	28 254
BNBC on ICE entries	1099 648	1571	215 792	100 342	27 533

**Figure 10:**
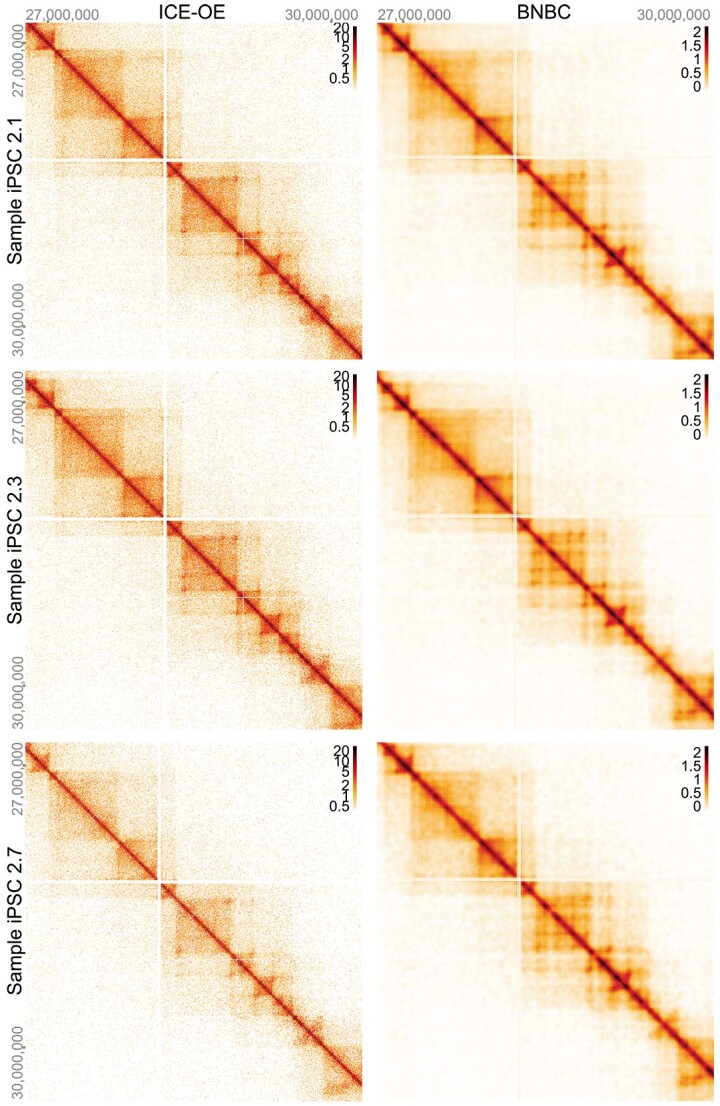
**Structure of Hi-C data following BNBC and ICE-OE.** Data are three samples from Greenwald *et al*. [50]. We display 3MB from chr22 at a 5 kb resolution. Data in the left column have been processed by ICE-OE, data in the right column by BNBC.

Finally, to establish that BNBC does in fact preserve local contact map features, we visualize a 3 MB region (at 5 kb resolution) of chr22 (chr22:27000000-30000000; Figure 10) using HiGlass [53]. To highlight finer detail (while allowing for clear visualization of some larger features) we zoom into a 2MB window (chr22:2800000-30000000), making the individual matrix cell contributions evident. While the results of ICE-OE and BNBC are technically on different scales, it is clear that both coarse and fine details are preserved by BNBC as compared with ICE-OE.

## DISCUSSION

Here, we have characterized unwanted variation present in Hi-C contact maps and the correction method BNBC. We show the existence of unwanted variation in Hi-C data and show that on average, experimental batch explains 32% of the between-sample variation in contact cells for ICE normalized data, in the 40 kb dilution Hi-C experiment analyzed here. We show unwanted variation exhibits a distance-dependent effect, in addition to known distance-based features of Hi-C contact maps. A simple combination of ICE and observed-expected normalization adapted to a between-sample normalization method corrects several of these deficiencies; we call this approach ICE-OE. We show that both ICE and ICE-OE has serious deficiencies when used for genetic mapping.

We present BNBC, a modular approach where we combine band transformation with existing tools for normalization and removal of unwanted variation for between-sample comparisons. This is not a method suitable if the intention is to pool data from different replicates into a single contact matrix. We show that BNBC performs well in reducing the impact of unwanted variation while still preserving important 3D features, such as the structure of the contact map and A/B compartments, as well as reproducible TAD calls. Data processed using BNBC show dramatic improvement when used for genetic mapping.

In the default mode, ComBat seeks to correct both the mean and the dispersion of the data that are attributable to batch. However, to accommodate users with batches which only have one replicate, ComBat can instead only correct the mean. This approach has been shown to correctly identify and correct batch effects [54], and we include this functionality for users for whom replicate level data are not possible. For gene expression analysis, models based on factor analysis such as remove unwanted variation [24, 28] or surrogate variable analysis [22, 23, 27] generally do not require batch labels or replicates and have shown outstanding performance. It will be useful to adapt such approaches to Hi-C data analysis.

We apply ComBat separately to each band, which allows for runtime to increase linearly in the number of bands to be normalized. A natural extension to ComBat would be a hierarchical model across bands; such a model would allow us to borrow information across bands. However, such a model would not have the same computational scaling properties as the setup we propose. We leave the development of such a model to future work.

As noted above, BNBC is designed for the setting where *a priori* observation of the same set of genomic interactions are equally probable across all samples, which is not the case for datasets exhibiting structural variation as is commonly found in many cancers. Additionally, because cancers from different individuals can exhibit different structural variations with differing copy numbers, explicitly modeling both variation and copy number will be critical for unbiased downstream analyses and such an approach has been successful in HiDENSEC [55]. Inference and integration of such information into our method would increase the flexibility of the model, but would necessitate a dramatic reduction in computational efficiency. The extension of this work to accommodate structural variation will be an important future development.

In terms of alternative methods, our approach is most readily compared with multiHiCcompare [31]. We find multiHiCcompare to be less effective in removing the variation in batch effect over distance relative ICE-OE in our assessments, and globally so when we compare with BNBC. We also observe that the data normalized by multiHiCcompare, but neither ICE-OE nor BNBC, enabled the discovery of chromatin loops.

As a by-product of both ICE-OE and BNBC we force the decay in contact probabilities over distance to be the same across samples. Haarhuis *et al*. [56] report that knockdown of WAPL in HAP1 cells results in changes in the contact decay probability. However, we show in our work that replicates can have quite different decay rates, which suggests that one should be careful before making claims about changes in decay rate. If decay rates are different across samples, forcing them to be similar will remove some biological signal and care should be taken with analysis.

Our approach does not employ the matrix balancing normalization, which is standard in Hi-C analysis. Matrix balancing is used to resolve confounders between different matrix entries from the same sample, such as GC content. Removing such confounders is important when comparing across matrix entries within a matrix from the same sample. In our work we are comparing individual matrix entries across replicates. Because we never compare different matrix entries to each other, this type of comparison is conditional on factors which depend on genomic location (such as GC content). This explains the seeming paradox of matrix balancing being widely used in single-sample Hi-C analysis, yet we found it to be insufficient for comparisons of individual matrix entries across samples. Additionally, this prevents the direct application of BNBC to data-derived samples where genomic structural rearrangements are to be expected, as is the case in many cancer samples. We leave the development of such a method for future work, and recommend the users to verify that their data do not exhibit structural variation prior to using BNBC.

We have found little reason to process the entire genome at once and have instead opted to process each chromosome separately. Given the potential for problems near chromosome arms where there is uncertainty of the length of the centromere, it may be desirable to process each arm separately. We note that our evaluation data on chromosome 22 are on a single arm.

We emphasize that our analysis of unwanted variation is about variation at the level of individual contact cells. The amount of unwanted variation can depend on the type of structure of interest such as TADs or loops. Our experiments suggest that BNBC enables the discovery of reproducible TADs, although ICE-OE may be more sensitive. At the same time, we note that neither BNBC nor ICE-OE enabled loop discovery.

In summary, proper normalization and correction for unwanted variation will be critical for comparing Hi-C contact maps between different samples.

### Author summary

Different loci of the same chromosome are assumed to interact with each other by the spatial folding of DNA. Such spatial interactions between different loci can be measured using Hi-C. Many Hi-C analyses focus on comparing different loci to each other, all measured on a single sample, and a number of single-sample normalization methods have been suggested to help with such analyses. However, there is increasing interest in making comparisons across different samples or conditions. We show that there is unwanted variation—also called batch effects—between different Hi-C experiments, likely of technical origin. Such unwanted variation is well studied for some genomic assays like RNA-seq, but are much less well appreciated for Hi-C data. We develop BNBC, a method for removing this unwanted variation, and show that using BNBC improves both a quantitative loci mapping analysis and a differential analysis between cell types.

**Key Points**

- BNBC corrects for unwanted variation in Hi-C data across samples.
- Methods such as observed/expected normalization or ICE do not correct this unwanted variation.
- Correcting this unwanted variation unlocks downstream analyses such as QTL studies.

## Supplementary Material

bnbc_r2s_over_distance_ns_bbae217

cors_over_distance_bnbc_ns_3batch_1_bbae217

Figure_r2other2_bbae217

frobenius_null_distributions_bbae217

frobenius_null_exemplar_bbae217

gm19238_mhc_rep1_contact_matrix_loops_bbae217

ld_21_hits_pur_yri_chb_LD_Matrix_bbae217

supplementary_bbae217

tads_ice_oe_bnbc_bbae217

## Data Availability

Compartments (Figure 6), QTL results (Figure 7) and differential enrichment results (Figure 9) are available from figshare, DOI: 10.6084/m9.figshare.c.5254002.

## Author Biographies

**Kipper Fletez-Brant** was a graduate student at the McKusick-Nathans Institute of Genetic Medicine at the Johns Hopkins University School of Medicine and the Department of Biostatistics at the Johns Hopkins University School of Public Health. His research interests include statistics and genomics and genetics.

**Yunjiang Qiu** was a gradate student at the Bioinformatics and Systems Biology Graduate Program at the University of California at San Diego. His research interests include bioinformatics and genomics.

**David U. Gorkin** was a postdoctoral fellow at the Department of Cellular and Molecular Medicine at the University of California at San Diego. His research interests include genetics, genomics and developmental biology.

**Ming Hu** is an associate staff at the Department of Quantitative Health Sciences, Lerner Research Institute, Cleveland Clinic Foundation. His research interests include Bayesian analysis in bioinformatics and statistical genetics.

**Kasper D. Hansen** is an associate professor at the Department of Biostatistics at the Johns Hopkins University School of Public Health. His research interests include methods and software for high-throughput genomics and genetics.
