# Supplementary figures and images for: Removing unwanted variation between samples in Hi-C experiments
Source: Brief Bioinform. 2024 May 6;25(3):bbae217. doi: 10.1093/bib/bbae217 (PMC11074651; doi:10.1093/bib/bbae217)

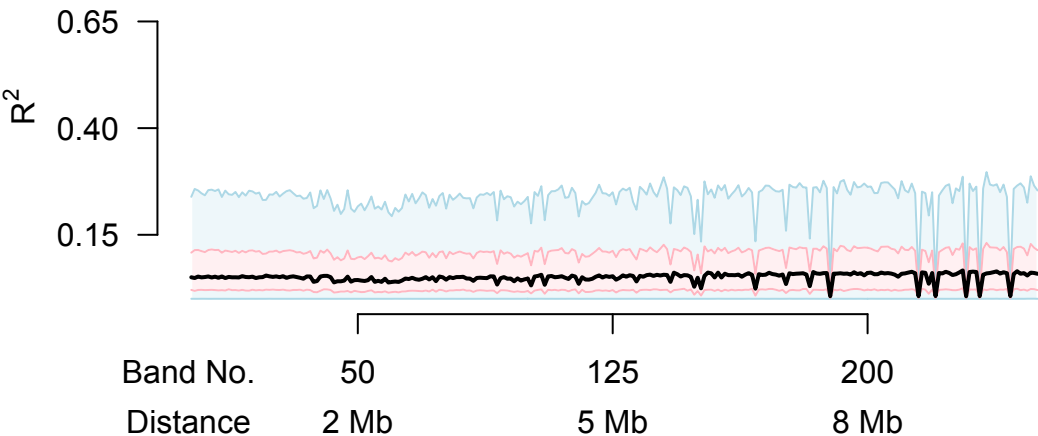

Supplement: bnbc_r2s_over_distance_ns_bbae217 [file bnbc_r2s_over_distance_ns_bbae217.pdf]

Correlation

0.5  
0.0

PC1 PC3  
PC2 PC4

Band No.

50

125

200

Distance

2 Mb

5 Mb

8 Mb

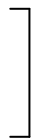

Supplement: cors_over_distance_bnbc_ns_3batch_1_bbae217 [file cors_over_distance_bnbc_ns_3batch_1_bbae217.pdf]

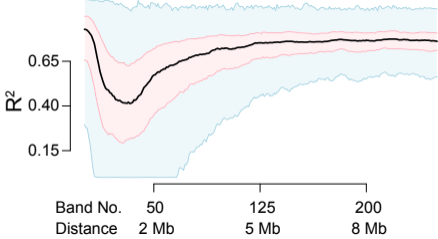

Supplement: Figure_r2other2_bbae217 [file figure_r2other2_bbae217.pdf]

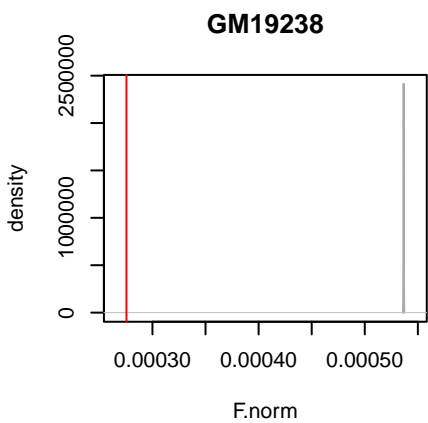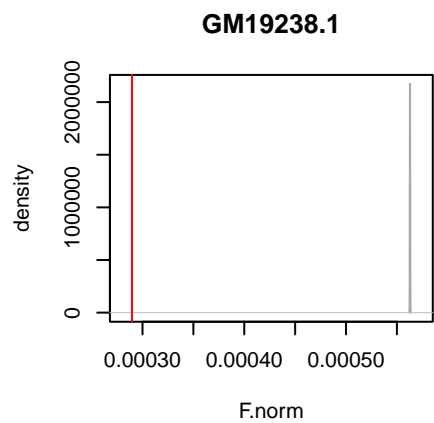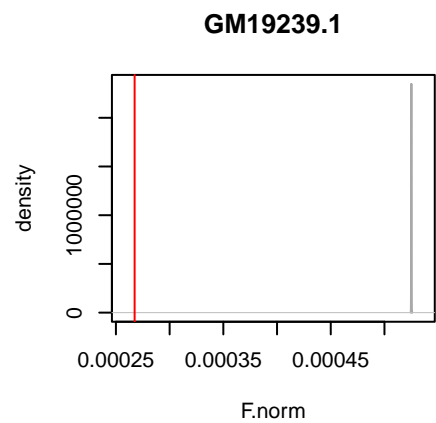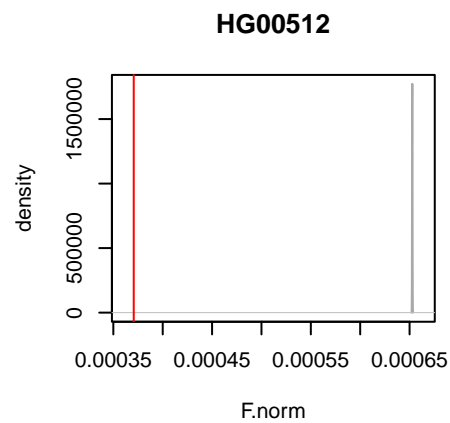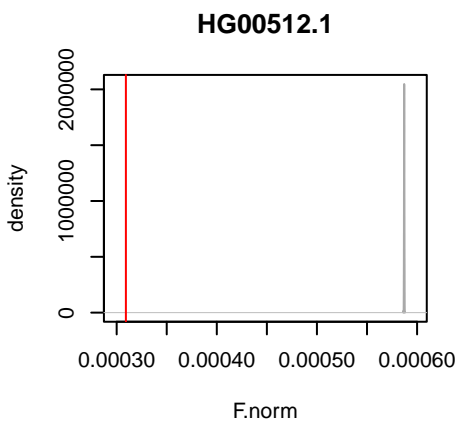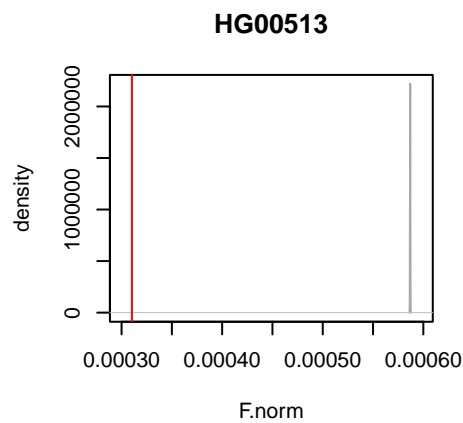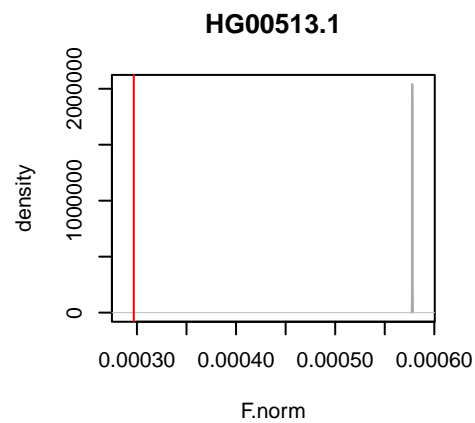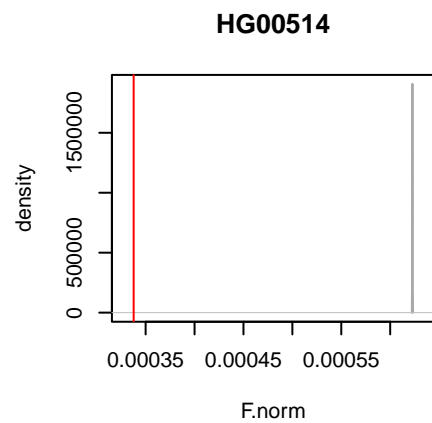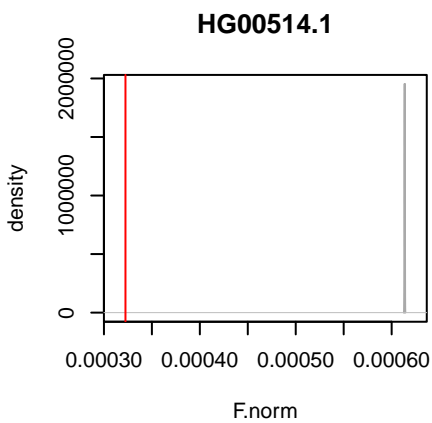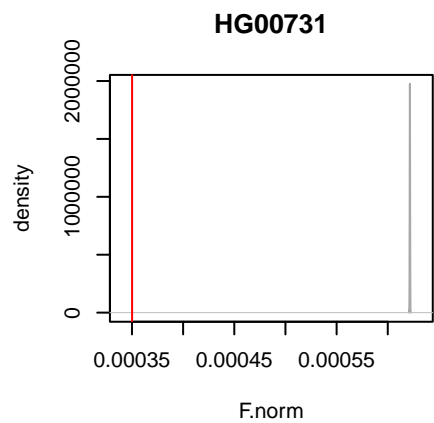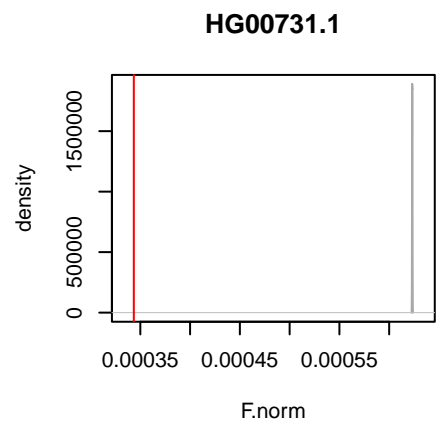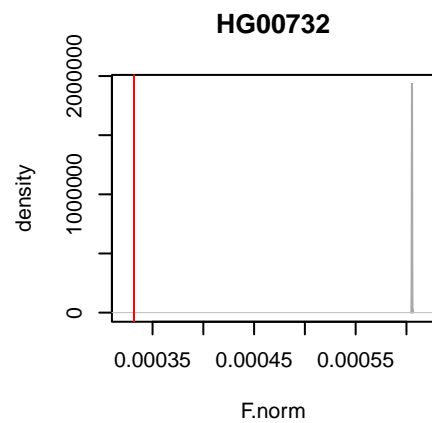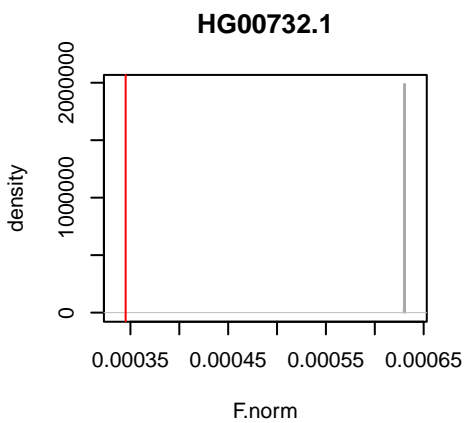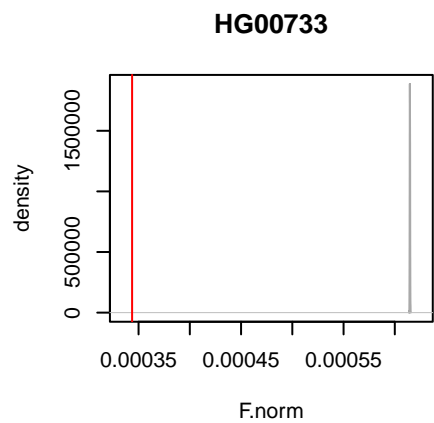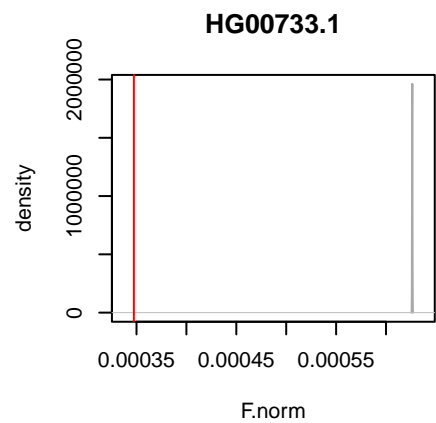

Supplement: frobenius_null_distributions_bbae217 [file frobenius_null_distributions_bbae217.pdf]

# GM19238 (inset)

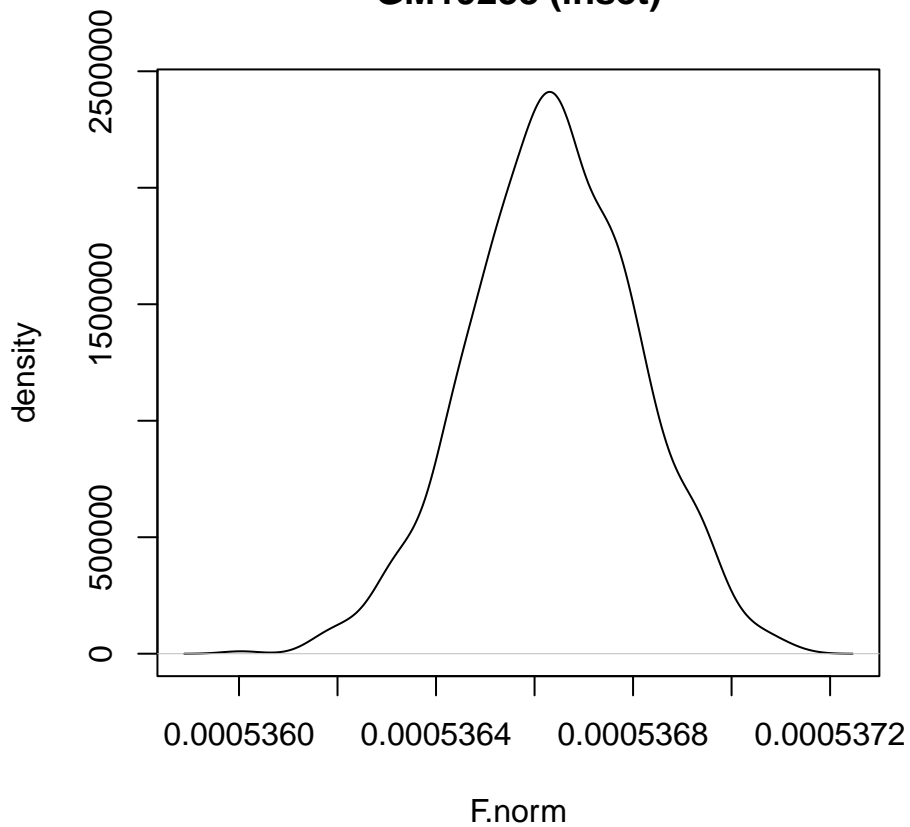

Supplement: frobenius_null_exemplar_bbae217 [file frobenius_null_exemplar_bbae217.pdf]

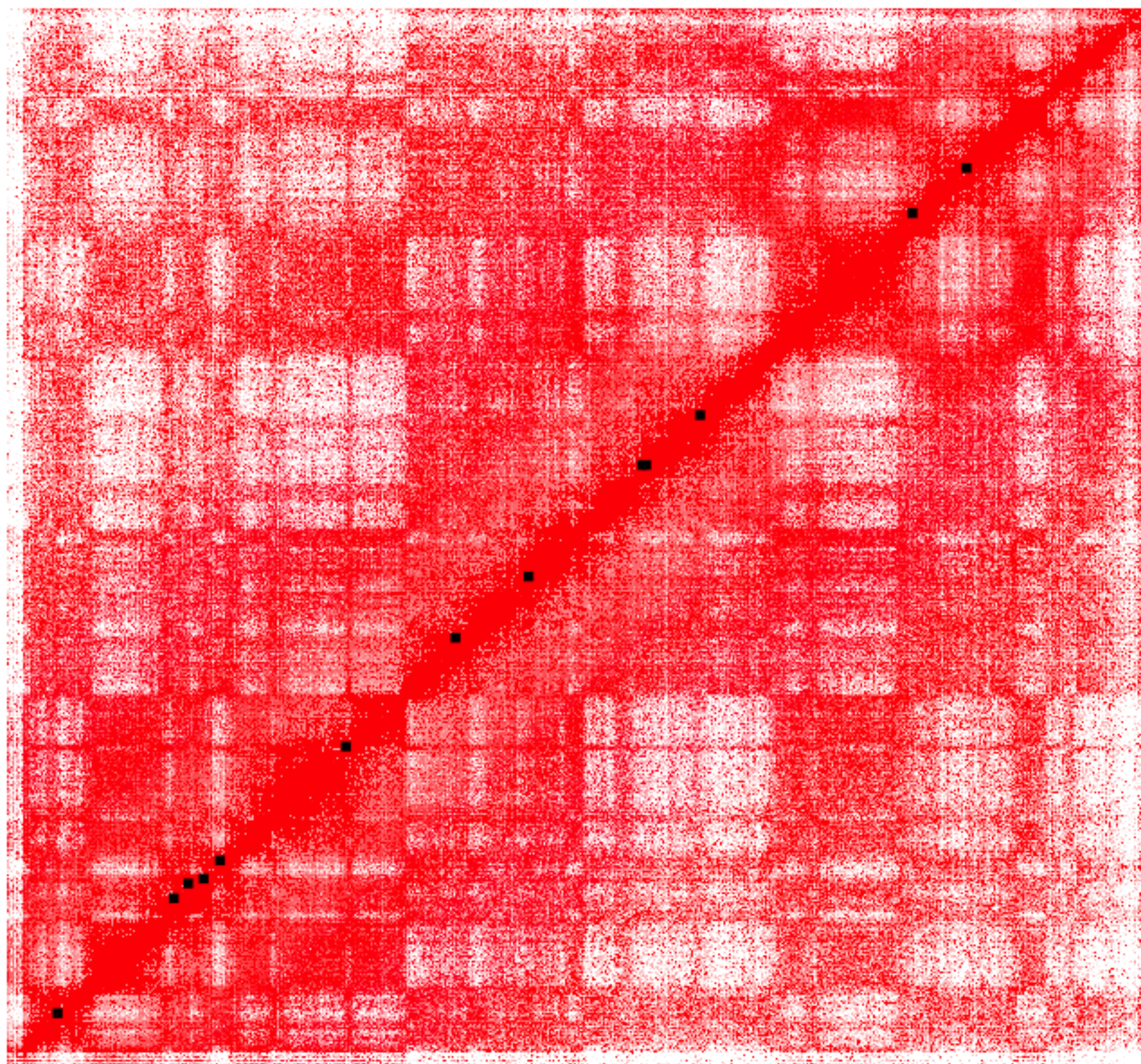

Supplement: gm19238_mhc_rep1_contact_matrix_loops_bbae217 [file gm19238_mhc_rep1_contact_matrix_loops_bbae217.pdf]

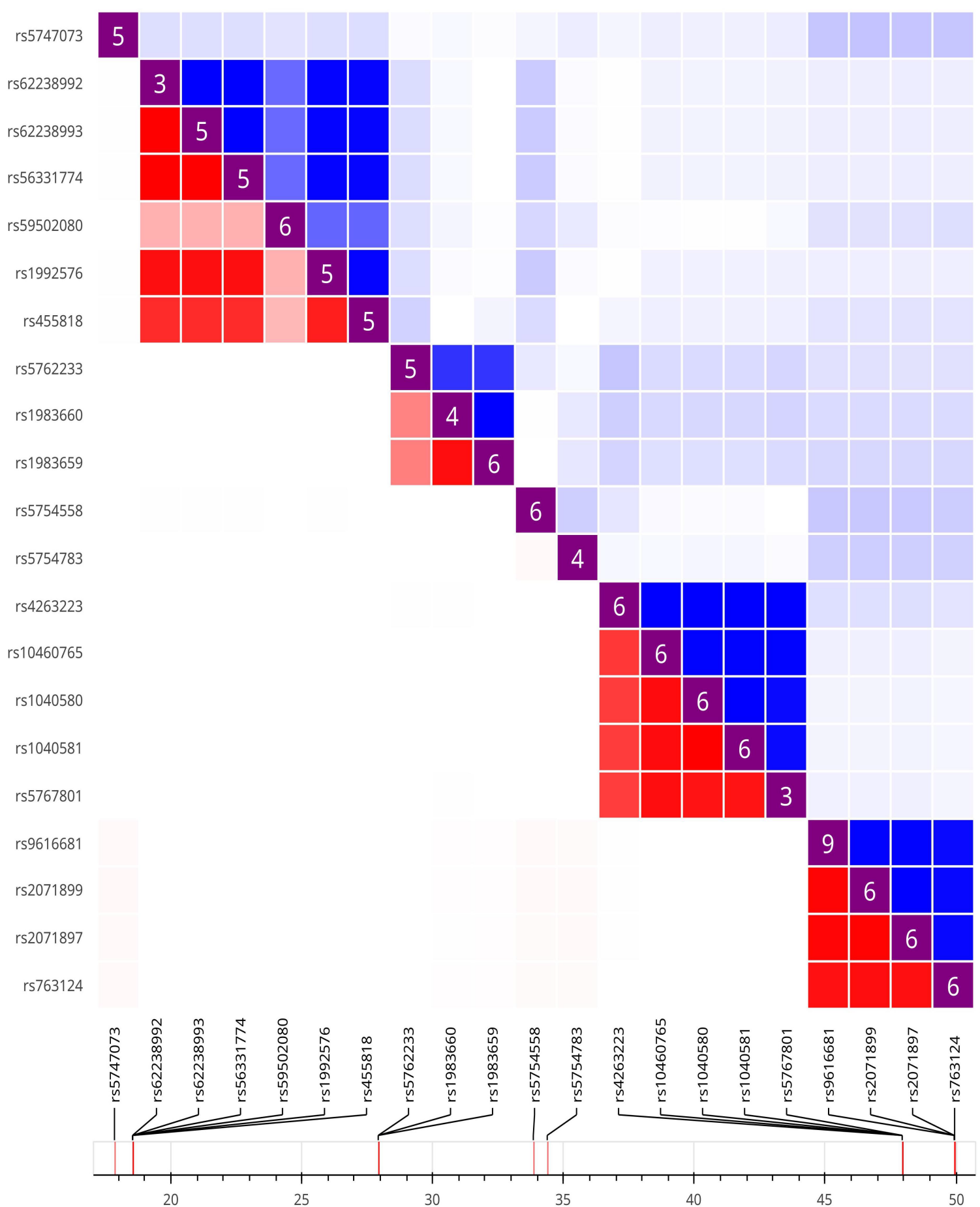

Supplement: ld_21_hits_pur_yri_chb_LD_Matrix_bbae217 [file ld_21_hits_pur_yri_chb_ld_matrix_bbae217.pdf]

**(a)**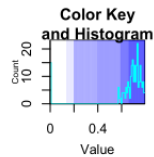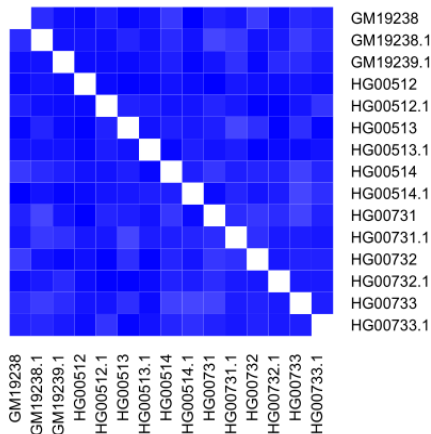**(b)**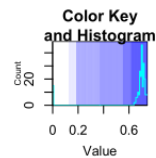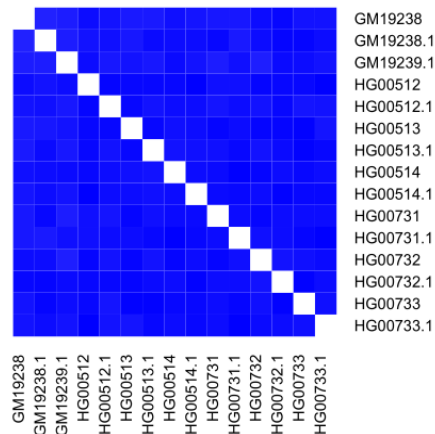**(c)**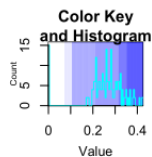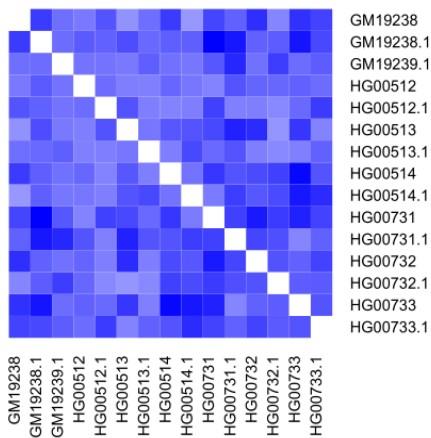**(d)**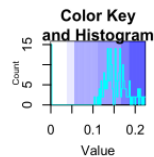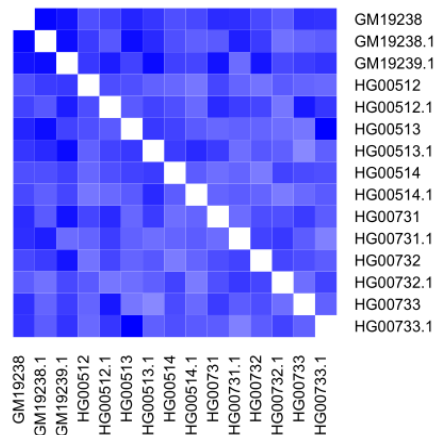

Supplement: tads_ice_oe_bnbc_bbae217 [file tads_ice_oe_bnbc_bbae217.pdf]
